# A Mobile App for Chronic Disease Self-Management: Protocol for a Randomized Controlled Trial

**DOI:** 10.2196/resprot.7272

**Published:** 2017-04-05

**Authors:** Raymond L Ownby, Amarilis Acevedo, Drenna Waldrop-Valverde, Joshua Caballero, Michael Simonson, Rosemary Davenport, Kofi Kondwani, Robin J Jacobs

**Affiliations:** ^1^ Nova Southeastern University Department of Psychiatry and Behavioral Medicine Fort Lauderdale, FL United States; ^2^ Nova Southeastern University College of Psychology Fort Lauderdale, FL United States; ^3^ Nell Hodgson Woodruff School of Nursing Emory University Atlanta, GA United States; ^4^ Larkin Health Sciences Institute Department of Clinical and Administrative Sciences College of Pharmacy Miami, FL United States; ^5^ Department of Instructional Design and Technology Fischler College of Education Nova Southeastern University Fort Lauderdale, FL United States; ^6^ Department of Community Health and Preventive Medicine Morehouse School of Medicine Atlanta, GA United States; ^7^ Baylor College of Medicine Department of Family and Community Medicine--Research Programs Houston, TX United States

**Keywords:** health literacy, chronic disease self-management, health disparities, patient activation, health related quality of life, mobile health technology

## Abstract

**Background:**

Health literacy is a critically important skill that helps people become active participants in their health care. Multiple studies in the United States and across the world have documented the association of health literacy with multiple health outcomes. In particular, the elderly and many members of minority groups have been shown to have low levels of health literacy; the same groups are disproportionately affected by chronic illnesses. These twin burdens affect the people most in need of the skills and knowledge required for coping with chronic illnesses. Chronic disease self-management (CDSM) is a logical target for a general health literacy intervention. In an approach that spans across specific diseases, CDSM targets problems and skills needed to cope with issues such as fatigue, pain, stress, depression, sleep disturbance, and treatment adherence. In a previous study, we showed that a computer-delivered tailored information intervention targeting health literacy could improve treatment and adherence and be cost effective, but it is not clear that this same strategy will be effective in persons with low health literacy and multiple chronic conditions.

**Objective:**

The purpose of this study is to develop a computer-delivered mobile intervention that will provide individuals with chronic conditions the necessary information to cope with their conditions.

**Methods:**

In this project, we will complete a qualitative study on the status and needs of individuals with more than one chronic condition. Results of this study will be used to develop a mobile tailored information app that will address self-management challenges in the areas of pain, sleep, fatigue, depression, anger, stress, memory problems, and treatment adherence. The impact of the intervention on patient quality of life, patient-provider relationships, health literacy, and patient activation will be assessed. We will also explore the extent to which health literacy mediates important outcomes, such as health-related quality of life and health service utilization.

**Results:**

We are currently completing the preliminary qualitative and usability studies that will inform the content and design of the intervention. We anticipate that the intervention will be complete in 2017, and the clinical trial of its efficacy will also commence in 2017.

**Conclusions:**

Results will provide evidence on the usefulness of a mobile tailored information app for improving health literacy, patient activation, health-related quality of life, and self-reported health in patients with multiple chronic conditions.

**Trial Registration:**

Clinicaltrials.gov NCT02922439; https://clinicaltrials.gov/ct2/show/NCT02922439 (Archived by WebCite at http://www.webcitation.org/6pTiqDAyN)

## Introduction

### Low Health Literacy and Disparities

Health literacy is a critically important ability that is a key route by which people can become active participants in their health care. The 2003 National Assessment of Adult Literacy in the United States showed that more than 75 million Americans had, at best, only basic health literacy skills, suggesting that as many as 1 in 4 are unable to understand the complexities of health care [1]. The importance of health literacy is underscored by multiple studies that have linked levels of health literacy to health status and outcomes [2-4] that even include risk of death [5,6]. Persons in racial/ethnic minorities and the elderly are even more likely to have low levels of health literacy. Twenty-four percent of blacks (9.5 million persons), 41% of Hispanics (21 million persons), and 29% of persons 65 years of age or older (12.5 million persons) have below-basic levels of health literacy [1], suggesting that they may be unable to use health information for even the most basic tasks, such as following simple directions on how to take a medicine. The ability to effectively obtain, interpret, and use relevant information to maintain health and cope with illness is increasingly important in today’s complex health care system, in which patients are required to take more responsibility for their care [7]. Further highlighting the importance of health literacy, compelling evidence links race and age to health via health literacy [3,8-12]. In the proposed study, we will assess the potential moderating effect of race, ethnicity, and age in response to an intervention to improve health literacy (Aim 3, discussed below).

### Need for Downstream Interventions Targeting Disparities

While the causes of health disparities are broad, complex, and rooted in social, cultural, and economic factors [13,14], health care services are still delivered to at-risk individuals every day. An urgent need exists for *downstream* interventions that can affect health disparities at the point of care [15]. Interventions that improve patients’ ability to manage their own health care may be especially critical given ongoing changes in the US health care system, which requires patients to take greater responsibility for their personal and family health care decisions [7,16,17], even as the system becomes more complex for underserved minorities [18] and the elderly [19]. Provisions of the Affordable Care Act in the United States, for example, specifically promote shared decision-making and the development of patient decision-making aids [20] to address these issues.

### Multimorbidity and Chronic Disease Self-Management

Chronic disease self-management (CDSM) is a logical target for a downstream intervention to address disparities and low health literacy. CDSM skills involve a wide variety of strategies that can be implemented by patients themselves to cope with chronic conditions. These strategies can include, for example, cognitive techniques for managing chronic pain (eg, distraction, reframing) or behavioral techniques (eg, graded exercise for fatigue and shortness of breath). While specific health conditions may require disease-related management skills (eg, glucometer use in diabetes), common issues such as fatigue, depression, stress, sleep disturbance, and treatment adherence span across illnesses and can be addressed in a single intervention; several of these have been the focus of a previously developed in-person group-delivered CDSM intervention [21-24]. In this continuation of our research on health literacy, we will expand on other interventions by first completing a qualitative study to inform the development of a general CDSM intervention. We will use the measure of health literacy created in an earlier study [25-27] to evaluate participants’ levels of health literacy and provide them with a version of the intervention tailored to their level of health literacy, preferred language, and race or ethnicity. In the proposed study, we will evaluate the effectiveness of a tailored intervention to improve health literacy (Aim 2, discussed more extensively below).

### Chronic Disease Self-Management and Health Literacy

CDSM integrates well with our previously developed and validated model of health literacy [26]. In the Abilities-Skills-Knowledge (ASK) model of health literacy, we argue that health literacy performances (such as finding and acting upon information about a disease) depend on general cognitive abilities (verbal reasoning, attention, working memory), specific skills (eg, reading and listening comprehension), and disease-related knowledge [26]. While efforts to improve basic cognitive abilities have been met with limited success, it is clear that patients can learn specific skills that will facilitate their understanding of oral and written health information, and can also learn conceptual knowledge about diseases. Created prior to the explicit formulation of the ASK model, the intervention targeting health literacy in persons treated for human immunodeficiency virus (HIV) [28-30] followed its principles and demonstrated the effectiveness of the instructional strategies discussed below. In this study, participants showed improvements in disease-related information and medication adherence [30]. An important finding in this study was that race-related differences in disease information were no longer significant after black participants completed the intervention [30]. In the project discussed in this paper, we will draw on this experience, as well as our current pilot study of an ASK model-based intervention for diabetes, in creating an efficacious intervention to improve health literacy in individuals with multiple chronic health conditions.

An alternate strategy to provide patients with the skills and knowledge to understand and manage their health is also important, because the dominant format for information delivery to patients in clinical care is oral communications during brief (and often rushed) clinical encounters [31]. Patient learning in these contexts is often suboptimal, at levels substantially less than 50% of the material presented [32], and during these encounters providers often fail to provide key information [33,34]. Unfortunately, it may be difficult or impossible to help patients develop self-management skills if they do not have basic health-related reading and math skills or health-related knowledge. Health literacy is likely to be an essential aspect of developing CDSM skills.

The ASK model will be the explicit basis for the development of our mobile app. We will first complete a qualitative study to better understand the skills and knowledge needs of individuals with both self-management needs and low health literacy. Results of this qualitative study will inform the development of the intervention. The ASK model will provide a general framework for the content of the intervention, while the results of the qualitative study will provide a guide to its content.

### Health Literacy Interventions

The previously developed conceptual model of health literacy [26] states that health literacy, after considering demographic variables, comprises basic cognitive *abilities*, relevant academic *skills* **,** and health care-related conceptual *knowledge* (ASK). An advantage of this model is that it leads directly to interventions. While it may be difficult to change patients’ basic cognitive abilities, interventions can readily be developed to improve patients’ skills at acquiring and understanding health information, along with their knowledge of health conditions and treatment. Strategies used in previous studies to improve patients’ health literacy have included providing information, improving skills (eg, reading comprehension), and targeting psychosocial constructs (eg, self-efficacy or patient activation) [3]. Reviews of these studies suggest that while some strategies were useful, no clear consensus on methods, content, or their effectiveness has emerged [3,8,35,36]. Expert recommendations for health literacy interventions include rejecting a *one size fits all* approach [37], creating interventions that promote participant engagement and retention through interactivity and interesting multimedia elements, and ensuring learning through an interactive teach-evaluate-reteach algorithm when needed [29,30,38,39]. The planned project will follow these recommendations in creating a tailored, interactive, and multimedia intervention to improve CDSM skills in participants with low levels of health literacy.

Tailoring content to make interventions more personally relevant and appropriate to patients’ levels of health literacy has been advocated as an effective strategy for health communication [40-42]. Tailoring can promote engagement and facilitate behavior change, as previously demonstrated in minority populations and those with low levels of education and computer skills [42-45]. Tailoring interventions to enhance racial and ethnic relevance enhances interventions’ effects for blacks [46,47] and Hispanics [48]. Other authors, including those who created national guidelines for the US Department of Health and Human Service’s *Healthy People 2020* and from the US Centers for Disease Control, stress the need to deliver materials that are matched to patients’ levels of health literacy [49-51]. Brouwer [52] noted that greater personal relevance may be related to increased likelihood of successful dissemination of computer-delivered health care interventions. Jerant et al [43] summarized research on computer-tailored health interventions and concluded that these interventions are a, “highly promising” strategy for reducing health disparities. Individualization of information and teaching strategies can be undertaken in small groups by skilled educators, but it is not clear how the health care system would find the number of educators needed or how they would be paid. Computer-based interventions can provide similar tailoring to larger numbers of patients at lower costs [39,43].

### Expected Outcomes of a Chronic Disease Self-Management Health Literacy Intervention

Effects of a successful health literacy intervention targeting CDSM are likely to go beyond improved knowledge and skills. Francis et al [53] showed, for example, that improving low-literacy patients’ reading skills also had a positive impact on their mood, and a study completed by our group showed that an intervention to improve health literacy in persons treated for HIV had a modestly positive (but nonsignificant) effect on control beliefs [29]. Hibbard et al [54] showed that an intervention to develop chronic disease management skills was associated with increases in patient activation, and Lorig et al [55] showed changes in patient self-efficacy for managing their health conditions after a chronic disease management program. During the evaluation of a health literacy intervention for CDSM, it will be important to measure not only health literacy as a specific outcome, but also to assess the effects of the intervention on other health care-related variables, including activation and utilization. In this study, we will also evaluate the effects of the intervention on problems that span across diseases, such as pain, sleep, stress, depression, and treatment adherence [56].

### Study Objectives

In a previous study, Fostering Literacy for Good Health Today/Vive Desarollando Amplia Salud (FLIGHT/VIDAS), we developed a new measure of health literacy [25]. The new measure was validated with respect to other measures of health literacy as well as health-related quality of life [27]. Data from the study were then used to posit and test the ASK model of health literacy, which could be directly linked to improved health literacy [26]. In the proposed study, we will adapt and deploy the FLIGHT/VIDAS health literacy measure on tablet computers (Aim 1, see below), use it to evaluate a participant’s health literacy, and feed this information forward to tailor the CDSM health literacy intervention to a level of health literacy that is appropriate for each participant. Data from the study will also be used to further explore the paths among race, ethnicity, age, health literacy, and health.

Although it has been shown that computer-delivered tailored information interventions may be effective in improving health literacy in specific patient groups [28,29,43,44,57], it is not clear whether the same sort of computer-delivered, multimedia, and interactive approach will be effective in improving CDSM-related health literacy skills in persons with low baseline levels of health literacy. If the strategy is effective, it is also not clear whether effects will extend beyond health literacy and target symptoms (eg, depression, pain, or adherence) to quality of life, self-efficacy, and patient activation. The effects of this intervention will be compared to an active control group consisting of a similar intervention presented without tailoring, and each group will be examined for effects on health literacy, health status, health self-efficacy, patient activation, and treatment adherence.

In addition to the overall goal of assessing the usefulness of a tablet-delivered app, this study will further several other project-related goals, including the ongoing development of the FLIGHT/VIDAS health literacy measure in its tablet adaptation. The utility of alternate forms of the measure and their sensitivity to intervention effects will be evaluated, and a final goal of the study is to use these data to explore the complex relationships among race, ethnicity, health literacy, and health status.

### Aims and Hypotheses

In Aim 1 we will adapt and evaluate the tablet computer-administered measure of health literacy developed during an earlier study with African-American, Hispanic, and white non-Hispanic individuals 40 years of age and older, which operated on tablet computers. The two hypotheses of Aim 1 are: (*Hypothesis 1*) alternate forms of the new health literacy measure’s general health literacy and numeracy scales will be sensitive to intervention effects, showing improvements after participants complete the intervention; and (*Hypothesis 2*) the new health literacy measure will be acceptable, usable, and valid when administered on tablet computers.

In Aim 2 we will use the computer-administered measure to assign participants to a racially- and ethnically-tailored intervention that is delivered in a way that is appropriate to their level of health literacy. The two hypotheses of Aim 2 are: (*Hypothesis 3*) an interactive multimedia intervention will be more effective in improving health literacy, self-reported health, patient activation, health self-efficacy, and treatment adherence compared to a matched active control intervention; and (*Hypothesis 4*) the interactive multimedia intervention tailored to participants’ characteristics will significantly reduce health literacy differences between white non-Hispanics, African-Americans, and Hispanics.

In Aim 3 we will assess mediators and moderators of the relation of health literacy to race and ethnicity. We will also explore how these mediators and moderators are linked to health behaviors and actual health outcomes. The two hypotheses of Aim 2 are: (*Hypothesis 5*) health literacy will mediate relations between age, race and ethnicity, socioeconomic status, cognition, academic skills, and health status; and (*Hypothesis 6*) health literacy will moderate response to the health literacy intervention, with blacks and Hispanics with lower initial levels showing a greater response to the intervention than white non-Hispanics with higher levels of health literacy.

## Methods

### Study Design

In Phase 1 of the study we will: (1) complete a qualitative study of critical chronic disease management skills and knowledge with key informants and patients, (2) use the results of the qualitative study to inform the development of a tailored computer-delivered CDSM health literacy intervention, and (3) complete acceptability and usability testing of the tablet-delivered version of FLIGHT/VIDAS and the intervention. In Phase 2, we will complete a clinical trial of the effects of the new intervention on multiple outcome measures.

### Phase 1

During the qualitative procedures in Phase 1, individual interviews will be completed with at least 20-30 individuals with a chronic disease (at least one chronic condition from planned inclusion criteria for Phase 2; sampling will continue until saturation is reached). Purposive sampling will be employed so that age, gender, race, language, and chronic conditions are adequately represented. During an earlier study, participants completed a questionnaire asking whether they had been diagnosed with specific health conditions. Results from this questionnaire will be used to recruit these same persons for Phase 1 procedures, based on the specific health conditions that were reported earlier. We will target individuals with a variety of conditions, as well as those with multiple conditions.

Interviews will be conducted in English or Spanish at Nova Southeastern University in Fort Lauderdale, Florida and at Emory University in Atlanta, Georgia, and information will be digitally recorded and transcribed for subsequent analyses. In-depth interviews will also be completed with 10 key informants (5 English-speaking and 5 Spanish-speaking) selected from diverse disciplines, who are familiar with the treatment of chronic illnesses, including: geriatric, internal, and family medicine; nursing; and social work. Interviews will be open-ended but will be semistructured to make it possible to elicit informants’ input on each of the key problems in chronic disease management that span across conditions. These problems include: fatigue, pain or physical discomfort, shortness of breath, sleep problems, depression, anger, stress, memory problems, and adherence.

After preliminary analyses, four focus groups will be completed (28 total participants, with one group in Spanish) with participants who are also representative of potential consumers of the intervention, to provide checks on concordance and trustworthiness of the themes identified in interviews. During these focus group sessions, we will solicit suggestions that more accurately represent the life situations of our study population.

Although interviews will cover the same material, interview content will be tailored to each participant's unique situation. Within each area, there will be several subareas designed to further understand these phenomena. We will use NVivo for Windows, Version 10 (Melbourne, Australia: QSR International) to assist in coding data, searching text, and conducting cross-case analyses. An inductive thematic analysis will be used, as this technique allows for the patterns, themes, and categories of analysis to emerge [58]. The software allows for a process that incorporates an emic approach to analysis, and allows for an exploration of indigenous concepts and typologies. The study design will allow for a consideration of the credibility of themes elicited in individual interviews by cross-checking them with focus group participants and key informants. In the final part of Phase 1, content modules will be refined and modified, based on this iterative process and using data from the interviews.

Intervention materials focusing on behavioral self-management skills will be created for each problem area (eg, exercise and self-guided cognitive behavioral interventions for pain [59], meditation for stress [60], behavioral activation for depression [61,62], cognitive behavioral interventions for sleep disturbances [63], and our own model for treatment adherence [29,38]). All key problems in chronic disease management listed above will be assessed by measures in our battery at baseline and all follow-ups, allowing for direct evaluation of the intervention’s effects on specific CDSM problems.

#### Interventions

Information from this qualitative phase will lay the basis for developing general content modules that are consistent with the ASK model of health literacy [26]. This process will draw specific content from established domains of chronic disease management by teaching health literacy skills and health condition conceptual knowledge.

Interventions will be developed that are consistent with recommended strategies for persons with limited literacy skills [64,65]. All materials will use health care-related content as a vehicle (eg, prose about diseases or a table about desirable blood sugars). Intervention elements will focus on the three basic literacy types, as defined by the Educational Testing Service: prose, document, and quantitative [66]. Consistent with research on the effectiveness of direct training in reading comprehension for adult learners [67], training in prose comprehension will focus on strategies to develop functional skills such as identifying key facts, inductive reasoning from multiple facts, and self-monitoring of comprehension. Training in document literacy will use tables, charts, and health-related forms (eg, insurance, consent) and emphasize strategies for identifying key information and strategies for using the information to answer relevant questions (eg, “Which insurance program is better for me?”). Training for quantitative skills will integrate basic instruction on quantitative concepts and procedures for understanding risk, probability, and cost [68,69].

#### Content

Information from the qualitative phase will allow us to develop general content modules that will focus on the key problems in chronic disease management listed above. Key strategies will be to teach health literacy skills and improve health condition-related knowledge. Problems, needed and useful skills, and disease-related conceptual knowledge revealed in qualitative work will then be used by investigators to create a content outline for each module. Content-relevant skills will be integrated with condition-related knowledge in each module (eg, by teaching reading comprehension skills using materials that are directly related to health). We will use a similar strategy in teaching listening comprehension by focusing on encounters with healthcare providers. This might involve teaching strategies for preparing a list of questions before an appointment, taking notes during the encounter, and being appropriately assertive in interactions.

Cognitive load theory [70,71] dictates that for optimal learning, instructional material must be presented in segments of content that do not exceed the learner’s ability to take in and retain each element. Consideration of this issue may be especially important in persons with limited health literacy [72]. Consistent with this issue, material will be presented in small segments via text and audio, with graphic elements reinforcing themes but not distracting from participants’ learning [70]. Interactions with the computer will only require that participants tap on the screen to press images of buttons, thereby keeping required computer skills to a minimum.

In a series of team meetings, content created by individual team members will be reviewed and alternate versions will be created at the three reading levels planned for each intervention group (3^rd^, 6^th^, and 8^th^ grade levels) based on the Flesch-Kincaid formula for English [73] and its Fernandez-Huerta modification for Spanish [74]. No clear criterion for the difficulty of health-related materials is available for use in tailoring, so we will use the criterion of grade equivalent levels. In this context, however, it will measure the difficulty of *health-related* materials and thus materials will be tailored to participants’ levels of health literacy.

Intervention content will be developed in either English or Spanish, with versions translated by team members fluent in the target language, with an emphasis on appropriate cultural adaptation rather than literal translation [75]. This strategy was previously effective in creating a health literacy measure in both English and Spanish [25], and helped to ensure that both language versions were intelligible to individuals from diverse linguistic and cultural backgrounds. Given the importance of regional usages of Spanish (eg, the same word may mean different things in Puerto Rico or Mexico), the Spanish version will be developed with an emphasis on standard Spanish to avoid comprehension problems. Intervention content will be assessed during usability testing, in addition to a focus on interface design and ease of use.

#### Usability Testing

After creation, acceptability and usability testing of the new materials will be completed using a procedure that we successfully used in other projects [76,77]. Individuals will be observed interacting with the computer app and will be asked to *think out loud* [78] so that their cognitive processes can be tracked, and areas of difficulty can be identified [76]. After five participants have completed this process, the intervention will be revised and tested again with a new group of five participants [77]. This process will be repeated until no further difficulties are identified, potentially eliciting responses from 15 to 20 participants [79,80].

#### Technology

Implementation will allow participants to monitor their own progress and provide centralized data collection. The intervention will be created in standard off-the-shelf software, *Captivate* (Adobe Corporation; San Jose, California), which is widely used in developing educational media. This software has been used in previous projects due to its ease of use, integration of multimedia elements, and simple deployment to most learning management systems. The current version (*Captivate 9*) creates apps in HTML5/CSS/JavaScript formats that can be readily deployed on Windows, iOS, and Android devices, and on the Internet. Output from this program can be readily packaged as downloadable apps for the operating systems of most mobile devices, including iOS (iPhone, iPad), Android, and Windows Phone.

#### Tablet Computer Adaptation

Delivery of FLIGHT/VIDAS on tablet computers will require modifications to the size of graphics, layout of page elements, and changes in font sizes. The file size for graphic elements will be reduced to improve download times over the Internet. Studies continue to show a dramatic increase in Internet use among older persons [81] and multiple studies show that elders evaluate the usability of tablet computers positively [82-84]. We will ask all participants to complete FLIGHT/VIDAS and the health literacy intervention on tablet computers, but it is possible that some of the oldest participants may not be able to do so. All participants in our previous study were able to complete assessments on touch screen computers (94 of whom were 70 years of age or older), so participants who have difficulty (ie, express frustration or state that they are not able to accurately record responses) with tablet computers will use the larger-format touch screen.

### Phase 2

#### Participants

**Figure 1:**
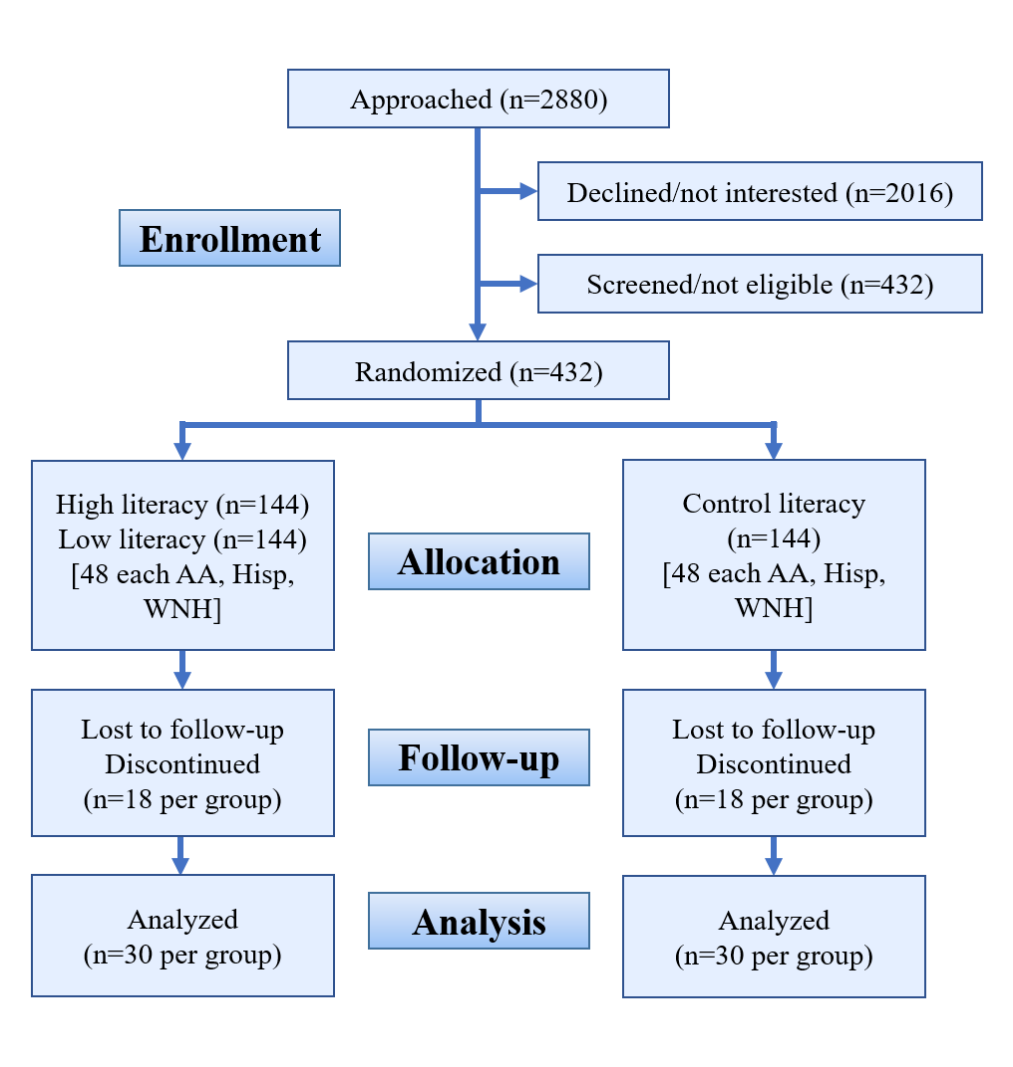
Patient recruitment and flow. Numbers reflect planned enrollment based on power analyses and potential attrition. AA: African-American or Afro-Caribbean; Hisp: Hispanic; WNH: white non-Hispanic.

We will recruit Spanish- and English-speaking persons aged 40 years and older whose health literacy is at the 8^th^ grade level or lower. Literacy levels will be assessed via validated scores on the Rapid Estimate of Adult Literacy in Medicine (REALM) [85], or the Short Assessment of Health Literacy in Spanish Speaking Adults (SAHLSA) [86]. This level was chosen to include persons in the lower end of the basic proficiency level and below, as defined by the US National Assessment of Adult Literacy [1,87-89]. The REALM and SAHLSA were selected based on their demonstrated relation to health status in other studies and data from our previous study that enabled us to use them as screening tools. The intervention will thus target those most likely to profit from instruction at a moderate difficulty level approximately two grade levels lower than this (6^th^ grade), or those who may need low-literacy level instruction (3^rd^ grade) based on their instructional reading level being approximately two grade equivalents below the tested level. English-speaking participants will include approximately equal numbers of African-Americans and non-Hispanic whites, with recruitment stratified by age decades (to enhance existing FLIGHT/VIDAS norms) from age 40, with no upper limit. Considering the importance of broadening the geographic basis of our normative population and ensuring the usefulness of the health literacy intervention for diverse groups, in the proposed study we will expand data collection to include Grady Memorial Hospital in cooperation with our collaborators at the Morehouse School of Medicine and Emory University (all in Atlanta, Georgia). We will continue to recruit African-American, white non-Hispanic and Hispanic participants at our Nova Southeastern University site, as we did in our earlier project. During this project, we plan to expand our recruiting efforts in South Florida to include larger numbers of Spanish speakers with low educational backgrounds who are originally from Mexico. We will randomize 432 participants, as needed (216 at each site; see power analyses for sample size justification, below). Numbers of participants in Figure 1 reflect planned recruitment based on power analysis and potential attrition.

### Treatment Assignment

#### Inclusion and Exclusion Criteria

Participants will be eligible for the study if they are 40 years of age or older, are treated for at least one of the conditions in the Functional Comorbidity Index [90] (cardiovascular disease, arthritis, cancer, lung disease, osteoporosis, depression, and others) with at least one medication, have health literacy below the 8^th^ grade level as indicated by the REALM (English) or SAHLSA (Spanish), and are willing to participate for the length of the study. Potential participants will be excluded if they are not able to provide informed consent, have a cognitive or psychiatric disorder that impairs their ability to safely participate in the study, or are not able to remain involved over the full length of the study.

A screening procedure for the new project was developed by assessing the ability of the REALM [85] or SAHLSA [91] to predict if participants had an 8^th^ grade or lower level of reading on the Woodcock-Johnson or Muñoz reading tests. Using the R statistical package pROC [92], a score of 62 on the REALM predicted reading skills less than an 8^th^ grade level on the Woodcock-Johnson (area under the curve [AUC]=0.79, 95% CI 0.72-0.87; sensitivity=0.80, specificity=0.67) with similar results for the SAHLSA. Earlier analyses showed that the FLIGHT/VIDAS General Health Literacy (HL) scale will also be effective for use in detecting participants with lower levels of reading skills (all *P-* values <.001). Analyses did not differ for language groups (AUC for Spanish sample=0.86, *P*<.001).

#### Recruitment and Retention

Recruitment at the Atlanta sites will take place in the Primary Care clinics of Grady Health Services and Emory Healthcare. Grady Health Services is the largest public health provider in Georgia, and fifth largest in the nation, with more than 130,000 outpatient visits per year. Emory Healthcare has satellite clinics throughout metro-Atlanta and serves a broad array of patient demographics and backgrounds. In current studies, it is possible to access the electronic medical record to prescreen study participants. We will continue this practice after obtaining Institutional Review Board (IRB) permission. The study recruiter will identify potential participants meeting inclusion criteria; he or she will then meet with clinic staff and providers, supply them with study information (flyers, brochures), and ask the clinic staff/provider to give the study information to these potential participants. Passive recruitment through study flyers and information about the study on clinic phone-wait systems will also be used. All studies utilizing these procedures are currently on target with recruitment goals.

In addition to a pool of subjects who participated in prior studies and have given permission to contact them in future studies, Spanish-speaking Hispanics will be recruited from Nova Southeastern University internal, family, and geriatric medicine clinics. Special efforts will be made to recruit a diverse group of Hispanics representative of South Florida and the rest of country’s Hispanic population, per US census data, particularly with respect to country of reference and educational attainment. In our previous study, we recruited more than 320 Spanish-speaking Hispanics over 3 years of active recruitment [25-28].

#### Interventions

Participants in the experimental groups will use modules that are tailored on three key dimensions: (1) preferred language (English or Spanish), (2) level of health literacy (3^rd^ or 6^th^ grade), and (3) racial or ethnic relevance (using verbal and graphic elements that will enhance the relevance of the material to African-Americans, Hispanics, or non-Hispanic whites). In the experimental groups, the intervention will add a fourth dimension of tailoring by providing individual feedback on understanding, via ongoing assessment of learning and review of material that is not yet mastered. The intervention content will be closely linked to the ASK model of health literacy [26] by targeting skills and knowledge related to CDSM, as informed by results of the qualitative study in Phase 1.

Participants in the control group will view informational screens and answer questions similar to the experimental group, but materials will be presented at the standard and widely recommended 8^th^ grade reading level (the approximate average reading level of adults in the United States [93]) to simulate the literacy demands that participants typically encounter when using health care information. Materials will not be tailored for race or ethnicity, except that Spanish speakers will complete the intervention in their preferred language.

#### What Will Participants Actually Do?

A typical intervention session will begin with the participant being greeted by study staff and directed to a touchscreen computer station. The study assistant will ensure that the patient has begun the correct study module (eg, self-management of sleep problems). The participant will view screens that present basic information on types of sleep disorders and their causes. After presentation of a discrete amount of information, a review screen is presented to consolidate learning (eg, “Common causes of insomnia are…”). The next several screens will present questions to check participants’ learning; if questions are answered incorrectly, the program loops to a review of the topic with a second presentation and check question. Specific sections of the intervention will focus on improving information search skills via computer-coached materials that facilitate comprehension of written materials on insomnia (eg, “What is the major point of this paragraph?”) and Internet information search skills (ie, how to find more information on the Internet). In addition, coaching on interacting with health care providers (eg, writing down questions before visits, or taking a friend with you who can help you remember what the doctor says) will be woven throughout the modules.

### Procedures

Participants will be recruited through advertisements, publicity at local clinics and senior centers, and presentations at local organizations. After initial contact, participants will be scheduled for a screening visit during which their eligibility for the study will be established via an interview to assess demographics, current treatment for at least one health condition, and level of health-related reading using the REALM or SAHLSA. During the baseline visit, participants will complete cognitive and psychosocial assessments and be oriented to the health literacy intervention. This visit will include baseline assessment of participants’ health literacy with Form A of the General HL and Numeracy (NUM) scales of FLIGHT/VIDAS. Participants will then be randomly assigned to the control or active condition, with assignment to low or high health literacy within the active intervention group based on HL scores. Participants in all groups will then complete three weekly visits (approximately 2 hours long) during which they will complete the health literacy intervention. All participants will thus complete 6 hours of the intervention. Final level of material attained will be recorded for use as a covariate in analyses, since some participants may progress more rapidly than others. At the end of the five weeks, participants will complete a postassessment visit during which they will complete Form B of the HL and NUM scales, along with the other outcome measures. Three months after this follow-up visit, participants will be asked to return for a final follow-up visit during which they will complete Form C (a combination of items from Forms A and B) of the HL and NUM scales and the same postintervention assessments of general health, activation, self-efficacy, and self-reported treatment adherence. At each follow-up visit, only measures hypothesized to be affected by the intervention will be administered. See Multimedia Appendix 1 for a detailed schedule of assessments.

#### Randomization and Blinding

After confirmation of eligibility, participants will be randomized to treatment condition according to a computer-generated schedule using the randomization procedures available in Research Electronic Data Capture (REDCap) electronic data capture tools hosted at Nova Southeastern University [94]. The principal investigator at each site (RLO, DWV) will be responsible for each treatment assignment.

#### Measures

Primary outcome measures will be health literacy (FLIGHT/VIDAS), self-reported general health (Medical Outcomes Study 36-item Short Form; SF-36 [95]), health self-efficacy (Chronic Disease Self-Efficacy Scale [96]), patient activation (Patient Activation Measure [97]), and treatment adherence. Additional data will provide the ability to consider race, ethnicity, age, and other variables as potential confounders. These data will include demographic variables, mood [98], stress [99], patient-provider relationships [100], treatment engagement, general cognitive abilities, academic skills, health conditions, current symptoms, health care attitudes, self-efficacy [101], and health care utilization.

Severity and number of health conditions will be assessed with the Functional Comorbidity Index [90], a previously validated measure linked to physical status, and an index based on the Midlife in the United States study [102] protocol, as used in our earlier study [27], to allow comparisons with data from that study. Assessments will include information on the specific symptoms of key problems in chronic disease management, and will allow a pre/postevaluation of the effects of the intervention on each. Simple measures of physical status (walking speed [103], body mass index [104], and waist-hip ratio [105]) will also be obtained to assess the effects of health literacy, cognition, and comorbidity on these variables, as they have all been related to risks for mortality [6,106,107].

Most self-report instruments will be administered via Automated Computer-Assisted Self-Interview (ACASI) software with assessment materials presented on touchscreen computers. This strategy enhances data collection by allowing participants anonymity in their responses and reduces issues with hand scoring of measures and manual data entry. Both sites will use the ACASI software Questionnaire Development System (QDS; Nova Research, Bethesda, Maryland). Individually administered measures, including assessments of cognitive, academic, and health literacy status, will be hand scored and verified. QDS output files will be transferred between sites using encrypted file transfer technology also available in REDCap.

The intervention will be standardized by its computer-based presentation across sites, but assessments and interactions with site workers must also be standardized to ensure fidelity across sites. Procedures will be standardized via visit flow sheets accompanied by training and monitoring visits by the investigators at each site. To ensure secure retention delivery of data across sites, all data will be entered into the REDCap software twice [94]. Each entry will be compared using utilities in REDCap and discrepancies will be resolved via inspection of original paper-based data collection instruments.

#### Data Safety and Monitoring

All assessment procedures planned in this study are either standard psychological or educational procedures (or are similar to standards), and all interventions are similar to typical educational interventions, so we believe that all interventions are associated with minimal risk to participants. We do not anticipate the need for a formal data safety monitoring board for this study, but we will follow a data safety monitoring plan. The plan will require ongoing monitoring of participants’ reactions to assessment and intervention activities with formal logs recording any events suggestive of participant distress, frustration, or boredom. All study personnel will be instructed on the importance of recording any relevant events in study logs, the contents of which will be reported on a monthly basis to the principal investigator for minor incidents, and immediately for any serious event (eg, substantial indication of participant distress such as crying, expression of desire to discontinue participation due to emotional upset, or request for emotional assistance due to of feelings or thoughts triggered by assessments). All events will be tracked and reviewed via regular conference calls with site principal investigators for determination of what changes in study procedures should be made to ensure the safety of participants.

Given the length of the study and frequency of study visits, we will be able to maintain close contact with all participants in an ongoing fashion. We will be able to develop and maintain a relationship with each participant that facilitates communication about their reactions to the study. Study personnel will be trained to be alert for signs of emotional upset or frustration in participants as a reaction to study procedures, and will be proactive in approaching participants about their emotional states. Participants will be offered supportive counseling if indicated, and referral for other services if needed.

Finally, we will routinely assess (both formally and informally) participants' reactions to the study using interviews and an ACASI questionnaire that will allow them to privately express any concerns or problems related to the study. We will actively monitor participants' experience in the study, both informally in exit interviews and formally via their responses to ACASI questionnaires, to determine if the study is excessively stressful or upsetting.

#### Informed Consent and Confidentiality

Informed consent is a process that will begin with IRB review of all recruitment materials. All study personnel will complete mandatory training in the protection of human subjects in research, including the courses of study contained in the Collaborative Institutional Training Initiative program [108], as required by the IRBs of Nova Southeastern University and Emory University, and only trained personnel will be involved in the process. The informed consent process will continue as study personnel explain study procedures during the education process preceding the request for written informed consent.

Participants will be protected from the risk of inadvertent disclosure of their health status or other health-related information by identifying all study-related data collection instruments by the participant's study number. Participants' identities will only be linked to data via a list that includes participant names and contact information and their participant identification number. All study databases will be kept on password-protected computers or secure servers (REDCap). Paper copies of all materials will be kept in locked filing cabinets in the investigators’ offices.

### Institutional Review Board Approval

Phase 1 (qualitative) study procedures were approved by the IRBs of Nova Southeastern University and Emory University. Phase 2 (clinical trial) study procedures have received preliminary approval by the IRBs of Nova Southeastern University and Emory University. Final approval is pending development and board review of actual intervention materials prior to their use with participants. Written informed consent will be obtained from all participants.

### Data Analyses

#### Initial Data Management

Initial review will evaluate data for completeness and valid characteristics through inspection of descriptive statistics. Although every effort will be made to minimize loss to follow-up, we recognize that some attrition is likely to occur. We will evaluate patterns of missingness in the data and follow widely-recommended procedures for dealing with this issue [109]. If our evaluation shows that missing data can be considered as missing at random (MAR) then our use of full-information maximum likelihood routines in MPlus statistical software (Muthén & Muthén; Los Angeles, California) will result in unbiased estimates of parameters [110]. If data are not MAR, we will include pattern of missingness in random effects pattern mixture analyses [111]. Group equivalence on demographic characteristics (except those defining group membership) and baseline measures not used in assignment will be assessed, and if differences are found these variables will be used as covariates in analyses. Other analyses will evaluate the distributional properties of dependent variables to assess the suitability of planned statistical models.

#### Study Hypotheses

Hypotheses will be tested via three aims, detailed below.

##### Aim 1

*Hypothesis 1* regarding the sensitivity of alternate forms of the health literacy measure to the intervention will be tested by calculating the simple sum of the HL and NUM scales for baseline (Form A) and first follow-up (Form B), and test their difference via paired *t*-tests, since this is a straightforward strategy that might be used in research and clinical work. This hypothesis will also be evaluated using a mixed-effects regression model with repeated measures for all three assessments that will include relevant covariates such as race/ethnicity, age, education, and cognition. Models for completers and intent-to-treat groups will be created. *Hypothesis 2*, regarding the validity of the measure on tablet computers, will be assessed by evaluating item difficulties and discriminations, calculation of scales’ internal reliabilities (Cronbach alpha), evaluation of construct validity via examination of the measure’s factor structure, and evaluation of concurrent validity as correlations and partial correlations of FLIGHT/VIDAS scales with other measures (Test of Functional Health Literacy in Adults, REALM, and SAHLSA). Measures of scale characteristics (mean, standard deviation, Cronbach alpha, and correlations with other measures) will be compared to values obtained for the full-size computer administration obtained during an earlier study [25] using tests for differences in means, standard deviations, and correlations.

##### Aim 2

*Hypotheses 3* and *4* for this aim, related to the effects of the intervention on outcomes, will also be evaluated in mixed-effects regression models including relevant covariates, with separate models assessing each dependent variable (HL and NUM scales, SF-36 General Health, Chronic Disease Self-Efficacy Scale, Patient Activation Measure, and treatment adherence) providing the ability to evaluate between-groups differences and the interaction of group membership (each racial/ethnic group). Estimated marginal means for mixed-effects models will be compared across levels of effects. Exploratory analyses will evaluate the effect of the intervention on other outcomes, such as patient-provider relationships, treatment engagement, and health care service utilization. Exploratory analyses will evaluate the impact of age on these outcomes. Similar analyses will evaluate the effect of the intervention on specific conditions, such as pain, sleep, and fatigue.

##### Aim 3

*Hypotheses 5* and *6* for this aim, related to the significance of effects of race, cognition, socioeconomic status, and academic skills on health status and utilities (as mediated by health literacy) will be tested using structural models developed in the MPlus statistical software. The significance of indirect (mediating) effects will be tested using bias-corrected bootstrapped estimates. *Hypothesis 6*, which suggests that levels of health literacy will moderate the effects of the intervention, will be tested by creating an interaction variable and including it in the structural model for *Hypothesis 5*.

#### Power Analyses

Power analyses were evaluated using procedures available in the software program *PASS 11* (NCSS, LLC; Kaysville, Utah). The effect sizes used were drawn from studies of reading interventions for low- and high-ability readers, and on the effects of our computer-delivered intervention on participant knowledge. Results show that a total sample size of 30 per group (high vs low vs control health literacy; African-American vs Hispanic vs non-Hispanic white), after possible attrition in any group of up to 38% (yielding at least 30 persons per group), will provide powers greater than 0.90 to test main effects and 0.80 or greater to test the interaction of time by group or by treatment condition. Power is adequate for all two-way interactions; the power to detect all three-way interactions would require a very large sample size that would be excessively costly. We will evaluate higher-level interactions even when not significant to assess the possibility of their presence.

### Expected Outcomes

Results of this study will help to establish the utility of the FLIGHT/VIDAS health literacy measure when administered on convenient tablet computers, including its validity in relation to other measures of health literacy and academic measure of reading and mathematics. Evaluation of the effects of tailoring strategies, and especially of providing information at a level appropriate for our participants’ level of health literacy, will provide a test of the usefulness of this strategy in general, and specifically as a way to test racial and ethnic disparities in knowledge and health status. Finally, mediation analyses will allow an assessment of the mediating effect of health literacy on health disparities.

### Dissemination and Sustainability

While not directly addressed in the study design, our development and evaluation strategy is focused on making the intervention more widely available in the long-term. A key aspect of this long-term strategy has been the decision to develop the intervention using a medium (HTML5/CSS/JavaScript) and responsive design strategy to ensure that the intervention can be deployed across operating systems and devices. In addition, while emphasis on responsive design will complicate the authoring and usability testing processes, this approach will help us to create an intervention that is widely usable across different devices and formats. The intervention can readily be made available over the Internet, and our development process also allows for packaging of the intervention as a downloadable app. Such apps can be posted for free download on mobile devices and be installed on commercial sites including the Apple Store, Google Play, and Windows store.

## Results

We are currently completing the preliminary qualitative and usability studies that will inform the content and design of the intervention. Analyses of these studies suggest that the planned intervention may be helpful to persons with multiple chronic conditions by providing information about management strategies. Results have also suggested the importance of spiritual and religious beliefs in coping with chronic diseases, and we are planning to incorporate this finding into the intervention. We anticipate that the intervention will be complete in 2017, and the clinical trial of its efficacy will also commence in 2017.

## Discussion

This study seeks to evaluate the efficacy of a mobile tablet computer intervention in targeting low health literacy directly, as a strategy to address health disparities. In a previous study of persons with HIV infections, the strategy of providing an interactive multimedia intervention that gave participants information tailored for personal relevance and level of understanding resulted in significant improvements in knowledge and treatment adherence [29]. In this project, we seek to build on this intervention strategy to specifically address the learning needs of individuals with low levels of health literacy. We will assess the impact of tailored interventions not only related to condition-specific problems, but also on broader outcomes such as health-related quality of life, patient activation, patient-provider relationships, and treatment adherence.

An important aspect of our development strategy will be the completion of a qualitative study that will inform the development of intervention content. Completion of usability testing, again with likely consumers, will help to ensure that the intervention will have a format and content that is likely to be useful to older persons with chronic conditions. It is hoped that this preliminary work will help to ensure the eventual acceptance of the intervention by a broader audience. The mobile format of the intervention will lend itself to use by persons who may not otherwise have been able to benefit from education on CDSM. While CDSM programs are provided in many locations, delivery of the programs in electronic formats may address some barriers (eg, rural residence, need to travel to a training site, and scheduling difficulties), especially for older persons who are employed.

Although it will be necessary for the dissemination of the intervention to follow development and the demonstration of its utility, it will be created with to the final goal of eventual widespread dissemination through the mobile app deployment system, and via the Internet. Another possible route of dissemination is for the intervention to be made available through patient portals that allow patients to access their health records. Most electronic health records that provide portals make provision for patient education, and the content developed in the planned study might be made available through this outlet as well. Providers would be able to prescribe that patients review material, thereby reducing the time spent in repetitive education activities, while facilitating a positive relationship with patients by providing the opportunity for interaction on patient questions.

## Appendix

Multimedia Appendix 1 Summary Statement from Previous Grant Reviews.

Multimedia Appendix 2 Summary Statement from Previous Grant Reviews.

## Abbreviations

ACASI: Automated Computer-Assisted Self-Interview
ASK: Abilities-Skills-Knowledge
AUC: area under the curve
CDSM: chronic disease self-management
FLIGHT/VIDAS: Fostering Literacy for Good Health Today/Vive Desarollando Amplia Salud
HIV: human immunodeficiency virus
HL: FLIGHT/VIDAS Health Literacy scale
IRB: Institutional Review Board
MAR: missing at random
NUM: FLIGHT/VIDAS Numeracy scale
QDS: Questionnaire Development System
REALM: Rapid Estimate of Adult Literacy in Medicine
REDCap: Research Electronic Data Capture software
SAHLSA: Short Assessment of Health Literacy in Spanish Speaking Adults
SF-36: Medical Outcomes Study 36-item Short Form
