# Supplementary material for: A Mobile App for Chronic Disease Self-Management: Protocol for a Randomized Controlled Trial
Source: JMIR Res Protoc. 2017 Apr 5;6(4):e53. doi: 10.2196/resprot.7272 (PMC5399224; doi:10.2196/resprot.7272)
Supplement: Multimedia Appendix 1 [file resprot_v6i4e53_app1.PDF]

Multimedia Appendix 1. Schedule of study procedures.

	Screening	Baseline	Intervention			Post	3 month
**Weeks**	-1	0	1	3	3	4	16
**PROCEDURE**			I1	I2	I3		
**INITIATION**							
Recruitment	X						
Telephone Assessment	X						
Informed Consent		X					
Allocation		X					
**ASSESSMENTS**							
Psychosocial							
Demographics		X					
SF36		X				X	X
Patient Activation		X				X	X
Self-Efficacy & Attitudes		X				X	X
Mood & Stress		X				X	X
Health Conditions		X					
Mental & Physical Activity		X				X	X
Treatment Adherence		X				X	X
Health care Utilization		X					X
Acceptability & Exit Interview						X	X
Physical Status							
Timed walk		X				X	X
Waist-hip ratio		X					X
Body mass index		X					X
Cognitive							
General cognitive ability		X					
Attention & executive		X					
Academic skills		X					

Health Literacy							
FV Form A		X					
FV Form B						X	
FV Form C							X
TOFHLA		X				X	
REALM	X						
SAHLSA	X						
**INTERVENTION**							
Introduction			X				
Fatigue			X				
Pain			X				
Sleep				X			
Depression				X			
Anger				X			
Stress					X		
Memory					X		
Adherence					X		
