# Supplementary material for: A Mobile App for Chronic Disease Self-Management: Protocol for a Randomized Controlled Trial
Source: JMIR Res Protoc. 2017 Apr 5;6(4):e53. doi: 10.2196/resprot.7272 (PMC5399224; doi:10.2196/resprot.7272)
Supplement: Multimedia Appendix 2 [file resprot_v6i4e53_app2.PDF]

SUMMARY STATEMENT
(Privileged Communication)

PROGRAM CONTACT:
Jennifer Alvidrez
301-594-9567
alvidrezjl@mail.nih.gov

Release Date: **05/19/2015**

Application Number: **1 R01 MD010368-01**

Principal Investigator

OWNBY, RAYMOND L MD, PHD

Applicant Organization: NOVA SOUTHEASTERN UNIVERSITY

Review Group: **ZRG1 HDM-X (56)**
Center for Scientific Review Special Emphasis Panel
PAR13-132: Understanding and Promoting Health Literacy

Meeting Date: **05/08/2015**
Council: **OCT 2015**
Requested Start: **09/01/2015**
RFA/PA: **PAR13-130**
PCC: **R01**
Dual IC(s): **NR, MH**

Project Title: **Health Literacy Assessment and Intervention to Reduce Disparities: FLIGHT/VIDAS II**

SRG Action: **Impact Score: 20 Percentile: 5**
Next Steps: **Visit http://grants.nih.gov/grants/next_steps.htm**
Human Subjects: 30-Human subjects involved - Certified, no SRG concerns
Animal Subjects: 10-No live vertebrate animals involved for competing appl.
Gender: **1A-Both genders, scientifically acceptable**
Minority: **1A-Minorities and non-minorities, scientifically acceptable**
Children: **1A-Both Children and Adults, scientifically acceptable**
Clinical Research - not NIH-defined Phase III Trial

Project Year	Direct Costs Requested	Estimated Total Cost
1	417,431	577,253
2	404,194	558,948
3	396,039	547,670
4	395,399	546,785
5	380,108	525,640
TOTAL	1,993,171	2,756,296

ADMINISTRATIVE BUDGET NOTE: The budget shown is the requested budget and has not been adjusted to reflect any recommendations made by reviewers. If an award is planned, the costs will be calculated by Institute grants management staff based on the recommendations outlined below in the COMMITTEE BUDGET RECOMMENDATIONS section.

RESUME AND SUMMARY OF DISCUSSION: This application requests support to develop and test a tablet-delivered, multimedia and interactive intervention to increase health literacy among older adults with low levels of health literacy who are diagnosed with chronic health conditions. If successful, the intervention will demonstrate significant improvement in patients' chronic disease self-management (CDSM) including general health status; self-efficacy and activation; and treatment adherence. The study has a high potential impact. In discussion, reviewers identified application strengths that include an accomplished team with diverse skills; use of health literacy measures that promise better precision; and novel use to technology to tailor intervention based on health literacy and knowledge of the patient. Reviewers also noted some minor weaknesses. These include concerns regarding the potential for attrition over the multiple sessions and the potential to arouse testing-related fear among participants. They also noted the need for more detail regarding assessment of health literacy across conditions. Overall this is an outstanding application with strengths that include the multi-skilled team and health literacy measurement strategy that far outweigh the noted minor, correctable weaknesses.

DESCRIPTION (provided by applicant): Health literacy is a critically important skill that helps people to become active participants in their health care. The 2003 National Assessment of Adult Literacy showed that more than 75 million Americans had basic health literacy skills, indicating that as many as 1 in 4 Americans can have difficulty understanding information about their healthcare. Persons in racial and ethnic minorities are likely to have even lower levels of health literacy. Twenty-four percent of blacks (9.5 million persons) and 41% of Hispanics (21 million persons) have below basic levels of health literacy. These persons have lower levels of health literacy and compelling evidence, including our own findings (see below), link race and ethnicity to disparities in health via health literacy. Members of minority groups and older adults are more frequently affected by chronic diseases such as cancer, high blood pressure, heart attack, stroke, diabetes, elevated cholesterol, asthma, hepatitis C, HIV infection, mental health disorders and many others. The twin burdens of chronic disease and low levels of health literacy thus fall disproportionately on those most in need – members of minorities and older adults, all of whom likely to experience one or more chronic conditions while often not having the health literacy skills to help them cope. Chronic disease self-management (CDSM) is a logical target for a general health literacy intervention. In an approach that cuts across specific diseases. CDSM targets problems and skills needed to cope with issues such as fatigue, pain, stress, depression, sleep disturbance and treatment adherence. Studies show that in- person CDSM classes improve patients' functioning and reduce healthcare utilization, but their availability is limited due to the lack of qualified personnel and cost. Similarly, while interventions have been developed to improve health literacy, they are difficult to scale to levels needed to meet the challenge of low health literacy (for more than 40 million persons) due to their cost. Effective interventions with the potential for wider dissemination at reasonable costs are urgently needed. In a previous study, we showed that a computer-delivered tailored information intervention targeting health literacy that can deployed either as an information kiosk in a clinical office or on the Internet could be cost- effective in improving patients' health literacy and adherence. It is not clear, however, whether the same sort of computer-delivered, multimedia and interactive approach will be effective in improving CDSM skills in persons with low baseline levels of health literacy, and if it is, whether its effects will extend beyond health literacy to general health, self-efficacy, activation, and treatment adherence. In this follow-up study we will evaluate this possibility by creating a personally relevant computer-delivered intervention targeting CDSM and health literacy among African-Americans, Hispanics, and white non-Hispanics:

PUBLIC HEALTH RELEVANCE: Improving patient health literacy through their chronic disease management skills and knowledge may be an effective strategy reducing health disparities. In this project we will build on our work in health literacy and computer-delivered interventions to create a new intervention to reduce health disparities by improving health literacy and chronic disease management among African-American, Hispanic, and non-Hispanic whites.

Critique 1

Significance: 1
Investigator(s):1
Innovation: 3
Approach: 2
Environment: 1

Overall Impact: Very strong team with a track record of expertise in the area who are adding new investigators as well. Focus on the intersection of chronic disease self-management, HL, and health disparities. Provides a compelling argument, conceptually based, on how the factors affecting health literacy also affect health outcomes. Thoughtfully developed educational modules based on the input from providers and those with chronic conditions. The multimedia modules are then tailored to the HL level and include health related content as well as instruction on improving HL. Outcomes are both proximal (e.g. HL) and distal (QOL, self-efficacy). Includes usability testing for the technology. Even if only the proximal outcomes are improved, the study is worth doing. Minor issues regarding analysis of dose effect (e.g. those who complete 2 sessions vs. 3), which is addressable. Strong environment.

1. Significance:

Strengths

- The intersection of health disparities, health literacy and multiple chronic conditions are well known. The proposed project attempts to address these issues with a computer-based chronic disease self-management approach.
- Using a HL tailored approach should have effects beyond the knowledge acquisition component as other studies have found additional effects.

Weaknesses

- None noted.

2. Investigator(s):

Strengths

- The PI, Dr. Ownby, has experience in the area of health literacy and technological adaptations. He has had other R01s. The remaining team members provide important expertise in HL among under-served populations, neurocognitive skills and HL, electronic learning, qualitative methods, programming and statistics. Members of the team have worked together in successful past projects. Thus team is strong, has complementary skills and should be able to complete the project as proposed.

Weaknesses

- None noted.

3. Innovation:

Strengths

- The proposed project extends and continues the prior work which has the potential to be widely used—e.g. tailoring the content for CDSM can be used in many other sites.

Weaknesses

- None noted.

4. Approach:

Strengths

- The intent is to evaluate HL using the investigator-developed instrument and then tailor the CDSM program using multi-media approaches. The investigators will examine whether the intervention affects HL, targeted symptoms that are generic to chronic conditions and extends into further effects (QOL, self-efficacy, patient activation). Even if the latter effects are not found, the test of the intervention is needed in these under-served populations.
- Extensive past work in the area although the preliminary studies section spends more time on the instrument development than on the test of the CDSM approach.
- Uses a learner-centered approach to identify the relevant life tasks required by the target groups with chronic conditions, thus responding directly to their needs vs. the usual investigator-centered approach.
- Intervention phase is reasonable for testing and will provide sufficient statistical power for the main effects.

Weaknesses

- High attrition (62%) anticipated—will there be analyses that compare completers with non-completers and by dose? E.g. if a participant does two of the three weeks of the intervention, is that sufficient to improve HL or QOL? The investigators will compare participants with refusal to participate and a similar approach would be informative for non-completers.
- The argument that a kiosk in a health center would be possible is not convincing—a two hour session using a kiosk in a health center would be a challenge for scheduling. Minor issue.

5. Environment:

Strengths

- Nova Southeastern provides the necessary infrastructure and support for the project to be successful.
- Emory, as a recruitment site, is well known for its strengths in research.

Weaknesses

- None noted.

Protections for Human Subjects:

Acceptable Risks and/or Adequate Protections

- Confusing paragraph about HIV diagnosis disclosure and IMB intervention--is this an inclusion from a previous study?

Data and Safety Monitoring Plan (Applicable for Clinical Trials Only):

Acceptable

- Uses a DSM plan, not a Board which is appropriate

Inclusion of Women, Minorities and Children:

- Sex/Gender: Distribution justified scientifically

- Race/Ethnicity: Distribution justified scientifically
- Inclusion/Exclusion of Children under 21: Excluding ages < 21 justified scientifically

Vertebrate Animals:

Not Applicable (No Vertebrate Animals)

Biohazards:

Not Applicable (No Biohazards)

Resource Sharing Plans:

Acceptable

- Very thorough

Budget and Period of Support:

Recommend as Requested

CRITIQUE 2

Significance: 1
Investigator(s): 1
Innovation: 1
Approach: 4
Environment: 1

<u>**Overall Impact:** This proposal addresses a significant issue applying innovative methods for health literacy education that have been developed by the team in previously research. If successful, the resulting computer-based program will have the potential to be implemented more widely and more cost-effectively than current programs which rely on in person training models in order to achieve tailoring of self-management strategies for individuals with chronic diseases. Another important feature of this project is that it is aimed at individuals with lower health literacy. There are some weaknesses in the approach; deployment of the program at Phase II is not yet worked out; generalizability may be negatively affected by those who avoid tests or have anxiety about learning programs/education; and there could be more detail about how literacy needs are assessed for different types of conditions and for those with multiple chronic conditions (which often require balancing different types of strategies).</u>

1. Significance:

Strengths

- Chronic disease and low levels of health literacy fall disproportionately on older adults and members of minority groups
- In-person educational and training programs are effective for promoting chronic disease self-management, but the intensity of the programs and supervision of personal training makes them difficult to scale up for broader implementation

- A computer-delivered tailored information intervention developed by these investigators would be a cost effective solution if it can be shown to have effectiveness among low literacy populations and minority older adults, using tablet computers

Weaknesses

- No concerns

2. Investigator(s):

Strengths

- Strong, qualified teams of investigators at both sites, with complementary skills
- Developers of the FLIGHT/VIDAS health literacy measure
- Have added new team members who have expertise in different aspects of the study design, e.g. cultural tailoring, qualitative research, and programming

Weaknesses

- No concerns

3. Innovation:

Strengths

- It is innovative to develop a system that will automatically generate materials for health education needs given the previous literacy and knowledge status of the person
- This can be done in person but to develop it using tablet based technology is likely to make the education program more implementable in more settings

Weaknesses

- No concerns

4. Approach:

Strengths

- The senior investigators are the developers of the FLIGHT/VIDAS health literacy computer-based health literacy measure
- Theory-based intervention plan based on their previous work and the Abilities, Skills, Knowledge framework
- Targeted at groups of lower literacy, racial minorities and older adults with chronic diseases
- Appropriate targeting and identification of issues that may affect chronic disease management: fatigue, pain, sleep problems, mental health, stress, memory, adherence
- Phase 1 to develop materials is appropriately qualitative (in-depth and other interviews); there are clearly stated analysis plans for these data
- Phase I interventions will be tailored for individuals at both group and individual level – language, individual level of health literacy, racial/ethnic group, and individual feedback for concepts not mastered. The control group will receive training at the 8th grade reading level with no specific tailoring (except that those who prefer Spanish will receive training in Spanish). Accommodations for the very oldest participants who might be unfamiliar with tablets. Phase I usability testing will be iterative (groups of 5 participants; total 15-20)

- Phase II will recruit a large sample in order to demonstrate potential for scalability; approximately 10 hours of training; pre-post design; intervention procedures will be standardized via flow sheets; well-developed analysis plan

Weaknesses

- More details would be helpful about how literacy needs are assessed for different types of conditions; participants with a wide variety of conditions will be included in the study. Do the skills generalize across all conditions? Will this be taken account post hoc if differences emerge? The tailoring is a great feature.
- Multi-session intervention in Phase II will have some practical challenges; likely will be deployed at point of care, which is consistent with the recruitment strategy for the sample, which mitigates the concerns somewhat. But patients often return at irregular intervals.
- Teaching and education often implies testing to many people and the antipathy of many adults to testing could be taken into account when evaluating the generalizability of this program in the population. Even if available in 1000s of practices in the future, would wider implementation require a motivational intervention as well? (Motivational interviewing has proven useful for enrollment in other self-management programs.)

5. Environment:

Strengths

- The research sites are more than adequate to support the scope of this project
- Coordination between the study sites is described (and the team has been in place for previously funded projects)
- Adequate recruitment sources

Weaknesses

- No concerns

<u>Protections for Human Subjects:</u>

Acceptable Risks and/or Adequate Protections

- Most procedures are minimal risk; feedback about cognitive screening and "testing" has the potential to be disturbing, but all of this feedback will be supervised and proper referrals will be made. Distress about procedures and feedback will be monitored.

Data and Safety Monitoring Plan (Applicable for Clinical Trials Only):

Not Applicable (No Clinical Trials)

<u>Inclusion of Women, Minorities and Children:</u>

- Sex/Gender: Distribution justified scientifically
- Race/Ethnicity: Distribution justified scientifically
- Inclusion/Exclusion of Children under 21: Including ages < 21 justified scientifically
- Equal numbers of men and women; good minority recruitment plan; the tool to be developed is not relevant to children.

<u>Vertebrate Animals:</u>

Not Applicable (No Vertebrate Animals)

Biohazards:

Not Applicable (No Biohazards)

Resource Sharing Plans:

Acceptable

Budget and Period of Support:

Recommend as Requested

CRITIQUE 3

Significance: 3
Investigator(s): 1
Innovation: 3
Approach: 4
Environment: 1

Overall Impact: This study proposes improve health literacy using a computer-delivered tailored information intervention system for chronic disease self-management (CDSM). The proposed tailored computer-delivered intervention targeting health literacy project could have a major positive impact on the health of individuals who have low general and health literacy skills and one or more chronic health issues by equip them with the ability and confidence to take a more active role in their health care. The tailored intervention and study design is strong and outweighs the noted weaknesses.

1. Significance:

Strengths

- Has potential to be a cost effective scalable strategy to reduce health literacy-related health disparities
- Public/private school collaboration Nova Southeastern/Emory is a strength

Weaknesses

- None noted.

2. Investigator(s):

Strengths

- PI and several co-investigators have secured grants and published regularly and extensively in this area:
- Diverse set of skills.

Weaknesses

- None noted.

3. Innovation:

Strengths

- Automated personal tailoring of intervention.

Weaknesses

- None noted.

4. Approach:

Strengths

- Three-phased design
- Detailed analysis
- Examines usability

Weaknesses

- Multi-session intervention in Phase II likely to be challenging, especially considering two-hour time blocks
- Not clear what will be done, if anything, to motivate participants to continue for the long haul

5. Environment:

Strengths

- Strong environment for PI and co-investigator organizations

Weaknesses

- None noted.

Protections for Human Subjects:

Data and Safety Monitoring Plan (Applicable for Clinical Trials Only):

Inclusion of Women, Minorities and Children:

- Sex/Gender: Distribution justified scientifically
- Race/Ethnicity: Distribution justified scientifically
- Inclusion/Exclusion of Children under 21: Including ages < 21 justified scientifically

Vertebrate Animals:

Not Applicable (No Vertebrate Animals)

Biohazards:

Not Applicable (No Biohazards)

Budget and Period of Support:

Recommend and Requested

THE FOLLOWING SECTIONS WERE PREPARED BY THE SCIENTIFIC REVIEW OFFICER TO SUMMARIZE THE OUTCOME OF DISCUSSIONS OF THE REVIEW COMMITTEE, OR REVIEWERS' WRITTEN CRITIQUES, ON THE FOLLOWING ISSUES:

PROTECTION OF HUMAN SUBJECTS (Resume): ACCEPTABLE

DATA AND SAFETY MONITORING PLAN (Resume): ACCEPTABLE.

INCLUSION OF WOMEN PLAN (Resume): ACCEPTABLE

INCLUSION OF MINORITIES PLAN (Resume): ACCEPTABLE

INCLUSION OF CHILDREN PLAN (Resume): ACCEPTABLE

COMMITTEE BUDGET RECOMMENDATIONS: The budget was recommended as requested.

NIH has modified its policy regarding the receipt of resubmissions (amended applications). See Guide Notice NOT-OD-14-074 at http://grants.nih.gov/grants/guide/notice-files/NOT-OD-14-074.html. The impact/priority score is calculated after discussion of an application by averaging the overall scores (1-9) given by all voting reviewers on the committee and multiplying by 10. The criterion scores are submitted prior to the meeting by the individual reviewers assigned to an application, and are not discussed specifically at the review meeting or calculated into the overall impact score. Some applications also receive a percentile ranking. For details on the review process, see http://grants.nih.gov/grants/peer_review_process.htm#scoring.

MEETING ROSTER

Center for Scientific Review Special Emphasis Panel
CENTER FOR SCIENTIFIC REVIEW
PAR13-132: Understanding and Promoting Health Literacy
ZRG1 HDM-X (56) R
May 08, 2015

CHAIRPERSON

BELGRAVE, LINDA LISKA, PHD
ASSOCIATE PROFESSOR
DEPARTMENT OF SOCIOLOGY
UNIVERSITY OF MIAMI
CORAL GABLES, FL 33146

MEMBERS

AMMERMAN, ALICE S, DRPH
PROFESSOR
CENTER FOR HEALTH PROMOTION
AND DISEASE PREVENTION
GILLINGS SCHOOL OF PUBLIC HEALTH
UNIVERSITY OF NORTH CAROLINA AT CHAPEL HILL
CHAPEL HILL, NC 27599

CAMPO, MICHELLE LYNN, PHD
ASSOCIATE PROFESSOR
DEPARTMENT OF COMMUNITY
AND BEHAVIORAL HEALTH
UNIVERSITY OF IOWA
IOWA CITY , IA 52242

DAVE, JAYNA MARKAND, PHD
ASSISTANT PROFESSOR
BAYLOR COLLEGE OF MEDICINE
SUGAR LAND, TX 77498

DIGNAN, MARK B, PHD
PROFESSOR
DEPARTMENT OF INTERNAL MEDICINE
PREVENTION RESEARCH CENTER
MARKEY CANCER CENTER
UNIVERSITY OF KENTUCKY
LEXINGTON , KY 40536

LIU, GILBERT C, MD
ASSOCIATE PROFESSOR AND DIRECTOR
DEPARTMENT OF PEDIATRICS
UNIVERSITY OF LOUISVILLE
LOUISVILLE, KY 40202

MADIGAN, ELIZABETH ANN, RN, PHD, BSN
ASSOCIATE DEAN FOR ACADEMIC AFFAIRS
INDEPENDENCE FOUNDATION PROFESSOR
BOLTON SCHOOL OF NURSING
CASE WESTERN RESERVE UNIVERSITY
CLEVELAND, OH 44106

MASTIN, TERESA
PROFESSOR OF PUBLIC RELATIONS AND ADVERTISING (PRAD)
COLLEGE OF COMMUNICATION
DEPAUL UNIVERSITY
CHICAGO, IL 60604

MILGROM, PETER M, DDS
PROFESSOR
SCHOOL OF DENTISTRY
UNIVERSITY OF WASHINGTON
SEATTLE, WA 981957475

SHAYA, FADIA TOHME, PHD
VICE-CHAIR FOR ACADEMIC AFFIARS AND DIRECTOR OF RESEARCH
PHARMACEUTICAL HEALTH SERVICES RESEARCH
CENTER ON DRUGS AND PUBLIC POLICY
SCHOOL OF PHARMACY
UNIVERSITY OF MARYLAND
BALTIMORE, MD 21201

VEINOT, TIFFANY CHRISTINE ELIZABETH, PHD
ASSOCIATE PROFESSOR
SCHOOL OF INFORMATION
UNIVESITY OF MICHIGAN
ANN ARBOR, MI 481091285

WETHINGTON, ELAINE , PHD
PROFESSOR
DEPARTMENT OF HUMAN
DEVELOPMENT AND SOCIOLOGY
CORNELL UNIVERSITY
ITHACA, NY 14853

MAIL REVIEWER(S)

LAMNERT, WILLIAM E, PHD
ASSOCIATE PROFESSOR
DEPT OF PUBLIC HEALTH AND PREVENTIVE MEDICINE
OREGON HEALTH & SCIENCE UNIVERSITY
PORTLAND, OR 97239

SCIENTIFIC REVIEW OFFICER

HENRY, REBECCA , PHD
SCIENTIFIC REVIEW OFFICER
CENTER FOR SCIENTIFIC REVIEW
NATIONAL INSTITUTES OF HEALTH
BETHESDA, MD 20892

EXTRAMURAL SUPPORT ASSISTANT

NJOKU, PHILIP C
CENTER FOR SCIENTIFIC REVIEW
NATIONAL INSTITUTES OF HEALTH
BETHESDA, MD 20892

Consultants are required to absent themselves from the room during the review of any application if their presence would constitute or appear to constitute a conflict of interest.
